# Lifestyle Intervention Guided by Group and Internet-Based Counseling in the T2D-GENE Trial Supports Its Applicability and Feasibility

**DOI:** 10.3390/nu15071787

**Published:** 2023-04-06

**Authors:** Ursula Schwab, Maria Lankinen, Matti Uusitupa, Markku Laakso

**Affiliations:** 1 Department of Endocrinology and Clinical Nutrition, Kuopio University Hospital, 70211 Kuopio, Finland; 2 Institute of Public Health and Clinical Nutrition, School of Medicine, University of Eastern Finland, 70211 Kuopio, Finland; 3 Institute of Clinical Medicine, Internal Medicine, University of Eastern Finland, Kuopio and Kuopio University Hospital, 70211 Kuopio, Finland

**Keywords:** diabetes, diet, human, intervention, lifestyle, physical exercise

## Abstract

Type 2 diabetes (T2D) can be prevented or postponed by lifestyle modifications as shown by previous intervention studies. In most of these studies, participants have received resource-demanding individual counseling. In the 3-year T2D-GENE trial with lifestyle intervention, we investigated whether a less resource-demanding form of group and internet-based counseling is feasible and effective in preventing T2D in people with an increased risk for T2D. Altogether, 628 middle-aged to elderly men either with a high number or low number of T2D risk alleles were recruited. Five to seven group sessions were organized during the intervention, in addition to information and activities delivered via the web portal, and weekly monitoring of body weight and physical activity. Four-day food records with personal feedback were documented five times during the study. Of the 549 participants completing the study, over 90% participated in the group sessions and kept the food records. The four self-feedback tasks delivered during the second and the third years of the study were completed by 80–89% of the participants. In conclusion, a group and web portal-based lifestyle intervention is applicable for middle-aged to elderly men as a lifestyle modification aiming to prevent T2D.

## 1. Introduction

Previous studies have shown that type 2 diabetes (T2D) can be prevented or postponed by lifestyle modifications, i.e., a health promoting diet, weight loss, and regular physical activity [1,2]. A systematic review and meta-analysis (SRMA) of the previous trials shows a 57% reduction in incident T2D [3]. In most of the previous intervention studies with lifestyle modifications, participants received individual counseling. From the resources point of view, this is a demanding task for healthcare. While the prevalence of T2D is rapidly increasing, it is not realistic to provide individual counseling to all or even a majority of patients at risk for T2D.

Genome-wide association studies have identified >400 common variants for T2D with small effect sizes, i.e., the risk of T2D is increased by 5–40% [4]. Most of the variants regulate insulin secretion, whereas only a few regulate insulin sensitivity. None of the previous intervention trials had considered the genetic background of the participants while recruiting participants. There are, however, some data from the Finnish Diabetes Prevention Study (FDPS) and the Diabetes Prevention Program (DPP) studies that suggest that lifestyle intervention may overcome the increased risk of some genetic polymorphisms, e.g., *TCF7L*2 and *PPARγ*2 genes [5,6,7]. In the FDPS study, *PPARγ*2 gene polymorphism significantly modified the efficacy of the intervention in body weight change and T2D incidence [5]. The TT genotype of the *TCF7L*2 gene has been reported to increase the risk of T2D in the control group but not in the intervention group in both the FDPS and the DPP studies [6,7]. A common variant of the *FTO* gene was not found to modify the effect of lifestyle intervention on body weight in the FDPS study [8].

In the T2D-GENE Trial, the primary aim was to compare the effect of lifestyle intervention (diet and physical activity emphasized) on the prevention of T2D and the worsening of hyperglycemia in people with a high number of T2D risk alleles compared with people with a low number of T2D risk alleles. Importantly, we also aimed to investigate whether a less resource-demanding form of group and internet-based counseling is as effective as individual counseling in preventing T2D in people with increased risk for T2D. The protocol of the T2D-GENE study has been previously described [9]. Here, we report on the method of the group and internet-based intervention in detail.

## 2. Materials and Methods

Altogether, 628 men were recruited from the Metabolic Syndrome in Men (METSIM) study as previously described [9]. The inclusion criteria were as follows: 1. IFG at entry (fasting plasma glucose 5.6–6.9 mmol/L) and with (2 h glucose 7.8–11.0 mmol/L) or without IGT (2 h glucose < 7.8 mmol/L), and HbA1c < 48 mmol/mol (<6.5%); 2. age 50–75 years; 3. body mass index (BMI) ≥ 25 kg/m^2^; and 4. the 1st or the 3rd tertile of the genetic risk score (GRS) (low risk or high risk, respectively). The GRS was calculated based on the number of risk alleles of 76 genes increasing the risk for T2D [10]. The tertile cut-off points were used to divide the participants into high or low risk GRS groups.

Participants in the intervention groups had four visits with laboratory samples (0, 1, 2 and 3 years) as previously described [9]. At the beginning of the intervention, there was a group session (about 90 participants at a time) where the aims of the study, and pathogenesis, complications of T2D, and potential of preventing T2D were presented. The participants were motivated to implement lifestyle modifications, as the importance of lifestyle modifications to reduce the risk of T2D was presented in detail during the session. In group sessions 2 and 3 (about 15–20 participants at a time), the participants were motivated further, and the principles of a health-promoting diet and physical activity were presented and discussed with the participants. The principles were based on the Nordic [11] and Finnish nutrition recommendations [12]. The principles of the European Guidelines for the prevention of T2D were also acknowledged [13].

Group session 2 included dietary fiber intake, consumption of fruits, vegetables, and berries, and intake of salt and sugar. Group session 3 included the quality of dietary fat, protein intake, meal pattern, and physical activity. Optimal dietary choices were presented from food groups, e.g., cereal products, dairy products, meat products, and dietary fats and oils. Those participants whose BMI was >28 kg/m^2^ had two additional group sessions concentrating on meal frequency, sources of energy, and the role of liquids, especially sugar-sweetened beverages and alcohol, as a source of energy. Eating behavior was considered as well, including, e.g., tips on how to overcome the desire to eat without hunger. For participants with BMI ≤ 28 kg/m^2^, these group sessions were voluntary. Regarding body weight, the minimum aim was to prevent weight gain during the study. Weight loss as such was not emphasized; the emphasis was on the health promoting diet and regular physical activity.

At 1- and 2-year group sessions, the participants were further motivated for lifestyle modifications, and the principles of a health-promoting diet and physical activity were repeated. Furthermore, new optimal choices on the market for a health-promoting diet were presented.

The spouses of the participants were welcome to all group sessions. Of the spouses, 30% participated in the motivation session and 16–22% in sessions 2 and 3. In weight loss sessions, the participation rate of the spouses was 14–15%. At the 1-year session, the participation rate of the spouses was 8%, and at the 2-year session it was 12%. Each session lasted about 90 min. The schedule, topics, and sizes of the groups are presented in Table 1.

Four-day food records were collected at baseline and at 6, 12, 24, and 36 months for dietary intake calculations (AivoDiet software, version 2.2.0.0, Mashie FoodTech Solutions Finland, Turku, Finland). Four-day food records were checked face-to-face upon being returned; these were checked by clinical nutritionists at all other time points except for at 6 months, when food records were collected via web portal or by mail and checked by phone. Each participant was given written detailed feedback on the results of the calculations, and, if needed, detailed tips were given to ensure participants were making optimal choices and reaching the aims set for dietary intake.

The participants were invited to use the web portal designed for the study. They reported their body weight and physical activity weekly via the web portal. Manual recording was possible for those participants who did not have access to the internet. Physical activity was reported in three categories, i.e., light, moderate, or heavy activity. The goal was brisk walking or other types of exercise with comparable intensity a minimum of 30 min per day at least five days a week.

**Figure 1:**
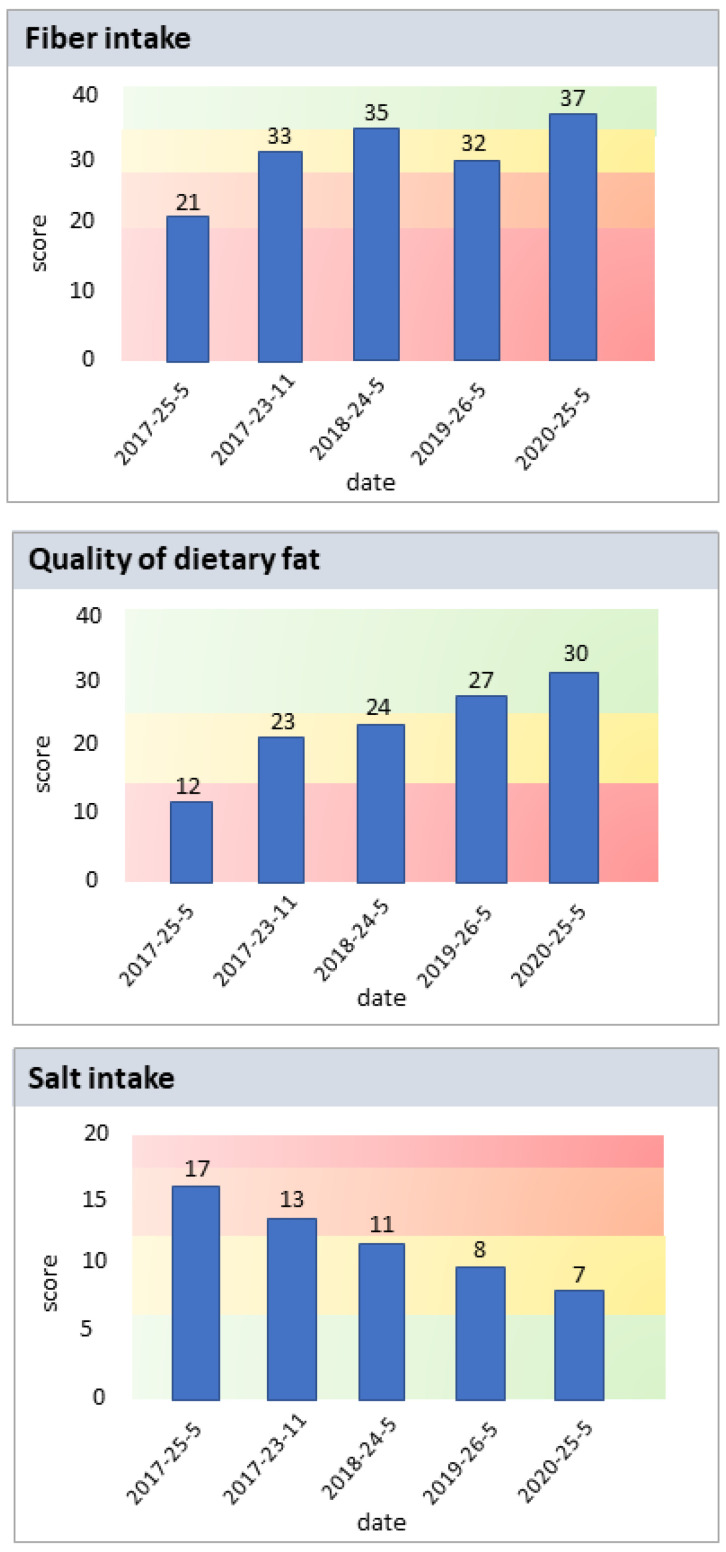
Examples of the results of the questionnaires filled by the participants on the web portal estimating their fiber intake, quality of dietary fat, and salt intake. The scores they obtained from the questionnaires were shown as these kinds of bar charts in the web portal. The colors illustrated whether their intake was optimal (green), fair (yellow and orange), or non-optimal (red).

The participants were also encouraged to fill simple, nationally-available questionnaires estimating their fiber intake, quality of dietary fat, and salt intake. Questionnaires on salt intake and quality of dietary fat were created by the Finnish Heart Association and the fiber test was modified from the version originally developed by the Finnish Bread Information Association (a general information service of bread business). The results were registered in the web portal, where the participants were able to follow their scores during the study (Figure 1).

There was also a discussion forum in the web portal, where the participants could present questions, comments, and obtain answers from the clinical nutritionists throughout the intervention.

Participants were contacted by the researchers monthly to provide information on health-promoting food choices and physical activity. In the materials, regarding the intake of certain food groups, e.g., fruits, some recipes suitable for a health-promoting diet were also included in the material provided. The topics of the monthly material are presented in Table 2.

**Figure 2:**
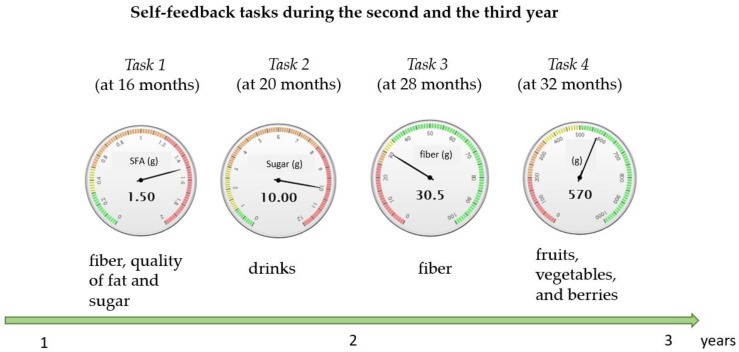
Self-feedback tasks during the second and the third years of the study. The participants indicated their typical food choices and tested what the effect is on their nutrient intake if they change their choices. The first task (month 16) included, e.g., choices of bread, spreads, and cold cuts, ingredients in fish soup, or coffee bread. The second task (month 20) compared sugar, fat, and energy content in different liquids. The third task (month 28) demonstrated the effect of choices in grains, vegetables, fruits, berries, nuts, and seeds on fiber intake. The fourth task (month 32) dealt with the intake of vegetables, fruits, and berries. Metrics indicated the amount of nutrients/foods, and colors illustrated whether the amount was optimal (green), fair (yellow and orange), or non-optimal (red).

During the second and the third years of the study, the participants were provided with four self-feedback tasks on the website. The topics were the following: dietary fiber, quality of dietary fat and sugar at 16 months, liquids and quality of fat at 20 months, dietary fiber at 28 months, and intake of fruits, vegetables, and berries at 32 months (Figure 2). At 20 months, the participants were also instructed to fill in a questionnaire on the quality of dietary fat on the website of the Finnish Diabetes Association. Those participants not having internet access were contacted by sending printed material by mail.

The participants were encouraged to contact the researchers anytime they had questions or concerns for virtual discussion with the clinical nutritionists. With those who did not have access to the internet, a phone was used for communication (phone calls and SMS).

## 3. Results

The 3-year intervention was completed by 549 participants. Of 628 participants who started the intervention, 26 developed T2D before the 3-year study visit and 53 discontinued the intervention due to, e.g., illness requiring treatment, or work-related limited time resources. The participation percentage in the group meetings among all participants was 92.0–99.5%. About 55% of the participants attended also the group meetings focusing on weight loss, which were primarily provided for those having a BMI over 28 kg/m^2^ (Table 1).

When the participants started, 97.6–99.5% of the participants completed the food records, whereas at the 6-month time point, the corresponding percentage was 92.6%.

**Figure 3:**
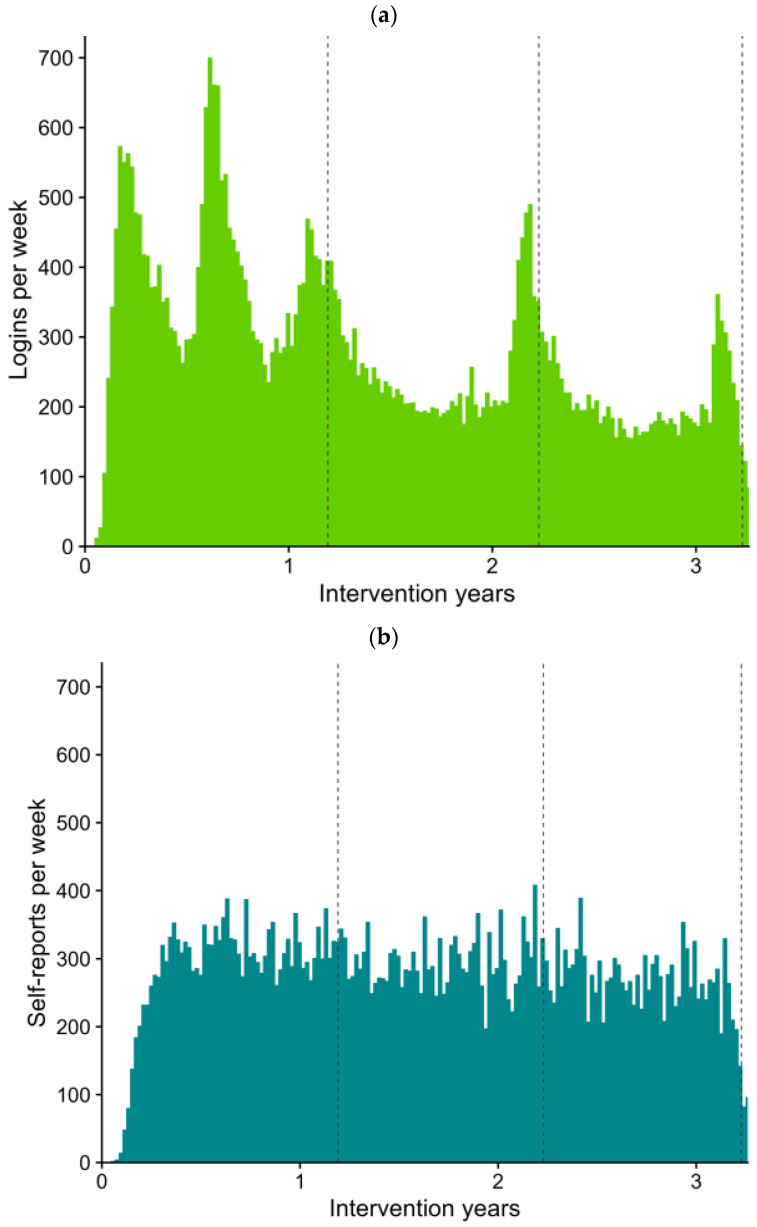
(**a**) Website logins during the intervention. (**b**) Weekly self-reports of physical activity (PA) and body weight (BW) during the intervention (reported at the same time). The dashed lines refer to the mean timepoint of the group sessions.

There were altogether over 51,000 logins to the web portal during the study, and 533 of the participants used the web portal at least once during the study. The web portal was used most actively during the first study year and there were peaks in the use close to the study visits, when food records were collected and when participants were asked to complete the tests for fiber intake, quality of dietary fat, and intake of salt in the web portal. The web portal activity is shown in Figure 3a.

Altogether, 560 participants recorded body weight and physical activity at least once during the intervention, either using the web portal or a written form (Figure 3b). The average recording times were 27 times per year, i.e., recording about every other week.

During the second and the third intervention years, the four self-feedback tasks provided on the website or via mail (Figure 2) were completed by 89, 89, 80, and 83% of the participants, respectively.

## 4. Discussion

A group and web portal-based lifestyle counseling intervention was very well welcomed by the participants based on their activity in participating in the group sessions, returning the food records, recording body weight and physical activity, and completing the tasks during the second and third years of the study, as well as through the login details on the website designed for the study.

The combination of dietary modification and physical activity has been shown to be an effective combination for T2D prevention [14]. Merlotti and coworkers [3] reported significant effectiveness in T2D prevention in their SRMA including 11 studies (8 RCT, 3 NRCT) with modifications in diet and physical activity (OR 0.43 (95% CI 0.35–0.52)). In most of the included studies, individual counseling was included. In the SRMA by Uusitupa et al. [2], including seven studies, a similar result was reported (RR 0.53 (95% CI 0.41–0.67)). All the included studies used individual counseling, either with or without group-based counseling and written instructions.

In the present study, the main emphasis was on both the quality of the diet and physical activity. In addition, healthy body weight, eating behavior, and alcohol intake were discussed in the group sessions. Zhang and coworkers [15] reported that dealing with several aspects of a health-promoting lifestyle is fruitful. Those having the healthiest lifestyle had a 75% lower T2D risk compared with those with the least healthy lifestyle, with no or only few features of a healthy lifestyle.

There are only a few studies reporting the results of a group-based intervention aimed at lifestyle changes. Pletsch-Borba and coworkers [16] found in the NutriAct intervention that in addition to the quality of diet at baseline, participation in group sessions resulted in higher compliance to the aims of nutrient intake. In a study by Moore et al. [17], a group-based counseling approach was reported to result in significantly improved diabetes knowledge, healthy eating, and physical activity levels in people with diagnosed prediabetes. In a Japanese study, a lifestyle intervention program combining individual and group counseling was beneficial in preventing T2D [18]. In one previous study in people with prediabetes, an automated behavioral intervention conducted by e-mail, web portal, and mobile phone resulted in favorable metabolic changes as compared with the control group [19].

One issue to be considered in dietary counseling is the profession of the healthcare professionals in charge of counseling. In the present study, clinical nutritionists designed the information provided in group sessions and via the web portal, as well as the tasks provided during the intervention. In an SRMA by Möller at al. [20], it was found that individualized nutrition therapy in people with T2D provided by a dietitian leads to a greater decrease in HbA1c, body weight, and LDL cholesterol concentration than dietary advice provided by other healthcare professionals. In a more recent SRMA, the results were similar [21]. In only a few of the previous intervention studies in people with increased risk for T2D, clinical nutritionists or dietitians have been in charge of dietary counseling. In the study by Sakane et al. [18], existing healthcare resources were used, including clinical nutritionists. In FDPS, clinical nutritionists were in charge of the dietary counseling as in the study by Penn et al. [22]. In the study by Bo et al. [23], nutritionists, specialists in endocrinology and internal medicine gave the instructions to the participants. So, in most of the previous intervention studies, healthcare professionals other than nutrition professionals were in charge of dietary counseling.

New approaches for the prevention of T2D are needed to manage the huge burden the healthcare system is facing due to the increasing number of individuals at risk for T2D. In addition to the high-risk strategy, population-based strategies are also warranted for the prevention of T2D. Distant counseling approaches might be useful also for lower income countries due to their lesser demand for financial resources. The approach used in the T2D-GENE trial retained the activity level among the majority of participants during the whole 3-year intervention and provided ideas for less resource-demanding actions for the healthcare system to meet the challenge of preventing T2D.

## 5. Conclusions

In conclusion, a group and web portal-based lifestyle intervention is applicable for middle-aged to elderly men as a lifestyle modification aiming to prevent T2D.

## Figures and Tables

**Table 1:** Timing, topics, and participation rates of the group meetings.

Group Meeting	Timing, Average Size of the Groups, Participation %	Topics
1: Motivation	Research months 1–3 60–90 participants/group 92.0%	Aims of the study, importance of lifestyle modifications on type 2 diabetes prevention
2: Healthy dietary pattern	Research months 1–3 15–20 participants/group 99.5%	Dietary fiber, intake of fruits, vegetables and berries, quality of carbohydrates, including sugar intake, salt, importance of lifestyle modifications on type 2 diabetes prevention
3: Healthy dietary pattern, exercise	Research months 1–3 20–30 participants/group 98.9%	Quality of dietary fat, protein intake, meal pattern, exercise, importance of lifestyle modifications on type 2 diabetes prevention
4: Weight loss I	Research months 3–6 15–20 participants/group 55.6%	Aims related to body weight, factors affecting weight, weight monitoring, meal pattern
5: Weight loss II	Research months 3–6 15–20 participants/group 53.8%	Eating behavior, liquids, tools for weight maintenance and weight loss
6: 1-year meeting	at 1 year 15–20 participants/group 98.3%	Fiber intake, fruits, vegetables and berries, fat quality, meal pattern, exercise, importance of lifestyle modifications on type 2 diabetes prevention
7: 2-year meeting	at 2 years 20–35 participants/group 90.7%	Findings from the study until 2 years, fiber, quality of dietary fat, importance of lifestyle modifications on type 2 diabetes prevention

**Table 2:** Topics of the monthly materials.

Topics
**Vegetables, fruits and berries:**
Fall season: Berries and apples
Fall season: Mushrooms and vegetables
Root vegetables
Tips for using vegetables, fruits, and berries
Seasonal vegetables and salad palette
Fruits
**Fat quality:**
Spreads
Nuts, seeds, and almonds
Cold cuts
**Grain products:**
Cereals, flakes, and mueslis
Fiber and salt in grain products
**Meal pattern and snacks:**
Meal pattern
Packed lunches for hiking and camping
Packed lunches and snacks for everyday life
Spoonable snacks
Dairy-based snacks
**Weigh control:**
Timing of meals, plate model, healthier choices as snacks
Liquids
**Seasonal:**
Christmas greetings with recipes
Summer greetings including recipes, e.g., for barbecue and lower fat ice cream products
**Exercise**
Health benefits of exercise
Group activities available in the region of the study site
Outdoor exercise
Everyday physical activity
Indoor activities and gym
Exercise in stairs
Light exercise, e.g yoga, stretching, outdoor activities
Strength and balance straining
**Others**
Ready-to-eat meals

## Data Availability

The data presented in this study are available on request from the corresponding author. The data are not publicly available due to privacy and ethical restrictions.
